# Differential expression of aldehyde dehydrogenase 1a1 (ALDH1) in normal ovary and serous ovarian tumors

**DOI:** 10.1186/1757-2215-3-28

**Published:** 2010-12-22

**Authors:** Krishna Penumatsa, Seby L Edassery, Animesh Barua, Michael J Bradaric, Judith L Luborsky

**Affiliations:** 1 Pharmacology, Rush University Medical Center, 1735 W Harrison Street, Chicago, IL 60612, USA; 2 Pathology Rush University Medical Center, 1735 W Harrison Street, Chicago, IL 60612, USA; 3 Obstetrics & Gynecology, Rush University Medical Center, 1735 W Harrison Street, Chicago, IL 60612, USA

## Abstract

**Background:**

We showed there are specific ALDH1 autoantibodies in ovarian autoimmune disease and ovarian cancer, suggesting a role for ALDH1 in ovarian pathology. However, there is little information on the ovarian expression of ALDH1. Therefore, we compared ALDH1 expression in normal ovary and benign and malignant ovarian tumors to determine if ALDH1 expression is altered in ovarian cancer. Since there is also recent interest in ALDH1 as a cancer stem cell (CSC) marker, we assessed co-expression of ALDH1 with CSC markers in order to determine if ALDH1 is a potential CSC marker in ovarian cancer.

**Methods:**

mRNA and protein expression were compared in normal human ovary and serous ovarian tumors using quantitative Reverse-Transcriptase PCR, Western blot (WB) and semi-quantitative immunohistochemistry (IHC). ALDH1 enzyme activity was confirmed in primary ovarian cells by flow cytometry (FC) using ALDEFLUOR assay.

**Results:**

ALDH1 mRNA expression was significantly reduced (p < 0.01; n = 5) in malignant tumors compared to normal ovaries and benign tumors. The proportion of ALDH1+ cells was significantly lower in malignant tumors (17.1 ± 7.61%; n = 5) compared to normal ovaries (37.4 ± 5.4%; p < 0.01; n = 5) and benign tumors (31.03 ± 6.68%; p < 0.05; n = 5). ALDH1+ cells occurred in the stroma and surface epithelium in normal ovary and benign tumors, although surface epithelial expression varied more in benign tumors. Localization of ALDH1 was heterogeneous in malignant tumor cells and little ALDH1 expression occurred in poorly differentiated malignant tumors. In benign tumors the distribution of ALDH1 had features of both normal ovary and malignant tumors. ALDH1 protein expression assessed by IHC, WB and FC was positively correlated (p < 0.01). ALDH1 did not appear to be co-expressed with the CSC markers CD44, CD117 and CD133 by IHC.

**Conclusions:**

Total ALDH1 expression is significantly reduced in malignant ovarian tumors while it is relatively unchanged in benign tumors compared to normal ovary. Thus, ALDH1 expression in the ovary does not appear to be similar to breast, lung or colon cancer suggesting possible functional differences in these cancers.

**Significance:**

These observations suggest that reduced ALDH1 expression is associated with malignant transformation in ovarian cancer and provides a basis for further study of the mechanism of ALDH1 in this process.

## Introduction

In previous studies we identified aldehyde dehydrogenase 1A1 (ALDH1) as a novel antigen in ovarian autoimmunity associated with unexplained infertility and premature menopause [1]. We also found that patients with ovarian cancer have anti-ALDH1 antibodies [2]. This prompted us to investigate the expression of ALDH1 in normal ovaries and ovarian tumors.

ALDH1 is a cytosolic isoform encoded by the *ALDH1A1 *gene at chromosome 9q21 [3]. ALDH1 belongs to the aldehyde dehydrogenase superfamily which is responsible for the oxidation of aldehydes to their corresponding carboxylic acids [4,5]. It is widely expressed during normal tissue development and homeostasis and is also found in immune cells [4-6]. Furthermore, ALDH1 expression is frequently altered in malignant tumors compared to their respective healthy tissues [7-10].

ALDH1 is responsible for tissue specific irreversible oxidation of retinal to the signaling molecule, retinoic acid (RA) [11]. RAs act through retinoic acid receptors and function in differentiation, reduced cell proliferation, tissue homeostasis and apoptosis in various cell types including ovary [12-17]. In ovarian cancer the expression of the retinol binding proteins involved in RA metabolism is reduced [18]. Also it was shown that in the intestine RA from dendritic cells imprints T and B cell homing, induces Treg cell differentiation [19,20] and induces tolerance [21]. This suggests ALDH1 and its product RA could influence tumor growth either through regulation of immune cells or by direct effects on tumor cell growth.

Moreb et al. using knock-down of the ALDH1A1 and ALDH3A1 genes in lung cancer cells showed that ALDH1A1 and ALDH3A1 accounted for cyclophosphamide resistance, cell growth and in addition affected other genes which have been implicated in cellular homeostasis and malignant transformation [22]. Recently, Deng et al. showed that increased ALDH1 expression was correlated with a chemo-resistant phenotype in ovarian cancer cell lines [7]. These findings suggest a critical role for ALDH1 in cancer and responses to drug treatment. Differences in tumor responses to treatment could be related to ALDH1 expression since it differs among different cancers [7] and is heterogeneously expressed among individuals for each cancer [23-25].

Aldehyde dehydrogenases are involved in steroid production, reproduction, oocyte maturation and early embryo development [26-29]. ALDH1 expression in normal human ovary and mouse ovary is among the highest compared to other tissues [30,31]. Inflammation is thought to be a predisposing event in malignant transformation [32]. Consistent with a possible modification of ALDH1 by inflammation, Rae et al. observed that exposing human ovarian cells to inflammatory stimuli resulted in down-regulation of ALDH1 [33]. Furthermore, ALDH1 expression is higher at early tumor stages [24,34] and may be correlated with clinical outcomes [7,24] in ovarian cancer.

In addition, studies in cancer stem cell biology revealed that ALDH1 enzyme activity can be used as a functional marker for isolating hematopoietic stem cells [35]. This has led to recent studies of ALDH1 as a marker in breast cancer stem cells [36]. The association of cancer stem cells (CSC) with ALDH1 in solid tumors has been shown primarily by its co-expression in cells expressing CSC markers [8,36,37]. This has not been investigated in ovarian cancer.

The high expression of ALDH1 in normal ovary, the established role of ALDH1 in detoxification and chemotherapy resistance and the potential role of ALDH1 in CSC in other tumors suggest that ALDH1 may have a significant role in ovarian cancer. There is little information on the relative expression of ALDH1 in human ovary and ovarian tumors. Therefore, to establish a basis for further studies on the mechanism of ALDH1 in ovarian cancer, we examined ALDH1 expression and localization in normal ovary and ovarian tumors in order to determine if ALDH1 expression is altered, if the cell types expressing ALDH1 changes and if ALDH1 expression in benign tumors resembles normal ovary or malignant tumors. We also examined the possibility that ALDH1 is co-expressed with the CSC markers CD44, CD117 and CD133 in order to determine if ALDH1 is associated with putative stem cells in ovarian cancer.

## Materials and methods

### Patients and tissue collection

Tissue was obtained from the Department of Pathology at Rush University Medical Center, Chicago, IL. All procedures followed an Institutional Review Board (IRB) approved protocol. Ovarian tissue was obtained from women with normal ovaries at hysterectomy (mean age 47.4 ± 3.4 years; n = 11), patients with benign serous ovarian tumors (mean age 56.1 ± 13.6 years; n = 9) and primary ovarian cancer patients with malignant serous ovarian tumors (mean age 58 ± 11.1 years; n = 8). The tumor histology and tumor grade were determined by diagnostic evaluation by a pathologist. Malignant serous tumors comprised Grade 3 (n = 6) and Grade 1 (n = 2) with Stage II (n = 3) and Stage III (n = 5) pathology. The criterion for inclusion in the study was women ≥ 40 years old (range 43-76 years; mean age 54.2 ± 11.6 years) and for the patients with benign or malignant ovarian tumors the inclusion criteria included primary serous ovarian tumors. The criteria for exclusion were previous history of any cancer and prior chemotherapy or radiation treatment.

### Assessment of mRNA expression

Total RNA was isolated using TRIZOL reagent (Invitrogen, Carlsbad, CA) according to the manufacturer's recommendation. RNA was measured at an optical density (OD) of 260 nm and the purity was evaluated using an OD 260/280 nm absorbance ratio ≥1.7. Before the first strand synthesis, 1 μg of total RNA was treated with DNase to remove trace genomic DNA. cDNA was synthesized using 500 ng of DNase treated RNA with a High-Capacity cDNA Reverse Transcription kit (Applied Biosystems, Foster City, CA) according to manufacturer's recommendation. Primer pairs were designed using Oligoperfect Designer software (Invitrogen) for *ALDH1A1 *[GenBank: NM_000689; in-between exon 6 and exon 7]. The Primer sequences were: ALDH1A1 Forward (5'- TTGGAATTTCCCGTTGGTTA-3') and Reverse (5'- CTGTAGGCCCATAACCAGGA-3'); Actin Forward (5'-CTGTGGCATCCACGAAACTA-3') and Reverse (5'- ACATCTGCTGGAAGGTGGAC -3'). The PCR amplifications were carried out in a 25 μl reaction volume containing 25 ng of cDNA using Platinum Taq DNA Polymerase (Invitrogen) according to manufacturer's recommendation. The mixture was denatured at 94°C (3 minutes) followed by 35 cycles at 94°C (30 seconds) and 54°C (30 seconds) to anneal and 72°C (1 minute) for extension followed by a final extension at 72°C (10 minutes) in a programmable Peltier Thermo Cycler (PTC-200, MJ Research Inc. Ramsey, MN). The PCR products were separated by electrophoresis in a 3% (W/V) agarose gel (Invitrogen) and visualized using ethidium bromide stain (Fischer Scientific, Pittsburg, PA). Amplicon from one positive sample each from normal ovary and ovarian serous carcinoma was purified using a QIAquick PCR purification kit (QIAGEN, Valencia, CA) and sequenced at DNA sequencing facility (University of Illinois at Chicago) using an ABI 3100 Genetic analyzer (Applied Biosystems). The amplicon sequences were blasted against the NCBI RefSeq human mRNA database and confirmed with a perfect match for *ALDH1A1 *gene [GenBank: NM_000689.3]. Quantitative Reverse Transcriptase-PCR (qRT-PCR) was carried out using SYBR green master mix in an ABI 7500 RT-PCR system and analyzed using the ΔCt method with human Actin as an internal control according to the manufacturer's recommendation (Applied Biosystems). The ΔΔCt was determined by subtracting ΔCt of each sample from the average ΔCt of normal ovary. The differences in ALDH1 mRNA expression levels were calculated as the fold change using the formula 2^-ΔΔCt ^as previously described [38].

### Immunohistochemical (IHC) detection of protein expression and localization

Tissues were fixed in formaldehyde, embedded in paraffin and sectioned (6 μm thick). Sections were mounted on microscope slides (Fischer Scientific, Pittsburg, PA), dried (16 hours; 37°C), deparaffinized in xylene, rehydrated in graded alcohols and rinsed with tap water. Sections were examined for histopathology following routine staining with hematoxylin and eosin (H&E; Sigma-Aldrich, St. Louis, MO). ALDH1, CD44, CD117 and CD133 expression was visualized using mouse anti-human ALDH1 mAb (clone 44, BD Transduction Lab San Jose, CA), mouse anti-human CD44 mAB (clone IM7; BioLegend, San Diego, CA), rabbit anti-human CD117 polyclonal antibody (C-19; c-Kit; Santa Cruz Biotechnology, Santa Cruz, CA) and mouse anti-human CD133 mAb (clone EMK08; eBioscience, San Diego, CA) respectively. Staining was carried out according to the manufacturer's protocol (Vector Laboratories, Burlingame, CA). In brief, antigens were unmasked by treating with antigen Unmasking solution (Vector Laboratories) and boiling in a microwave. Endogenous peroxidase was inactivated using substrate (0.3% H_2_O_2 _in methanol; 20 minutes; 22°C). Sections were washed with phosphate buffer and non-specific binding sites were blocked with normal horse serum (30 minutes). The sections were then incubated with mouse anti-human ALDH1 antibody (1:200) diluted in phosphate buffer containing 1% Bovine Serum Albumin (BSA; Sigma-Aldrich, St. Louis, MO) in a humid chamber (2 hours, 22°C). The bound anti-human ALDH1 antibody was detected using ABC Universal kit and the antigen-antibody reaction was visualized with 3, 3-diaminobenzidine peroxide substrate (DAB; brown color). As a control for secondary antibody binding directly to sections, the ALDH1 antibody was omitted. Sections were briefly rinsed in water, counterstained with hematoxylin (Fischer Scientific) and rinsed in running water (15 minutes). Double label immunostaining was carried out according to the manufacturer's multiple labeling protocol (Vector Laboratories). In brief, the ALDH1 stained sections were further treated with normal horse serum (30 minutes) to block non-specific binding sites. Sections were then incubated with anti-human CD44 or CD177 or CD133 antibody (1:100, diluted in 1% BSA in phosphate buffer) and processed as described for anti-ALDH1 alone, except that the color was developed with DAB and Nickel peroxide substrate (gray/black color). Finally, the sections were dehydrated in graded alcohols and xylene, and covered using Permount (Fischer Scientific). Sections were examined by light microscopy (Olympus BX-41, Center Valley, PA) and images captured and evaluated with MicroSuite Five software (Olympus).

### Semi-quantitative Immunohistochemistry

ALDH1 protein expression and localization was assessed using a unbiased cell counting stereology method with a microscope (Olympus BX60, Center Valley, PA) interfaced with a digital camera (CX9000; MBF Bioscience Williston, VT), motorized stage and image analysis software (StereoInvestigator 8.1, MBF Bioscience, Williston, VT). Cell estimation was performed using optical fractionator procedure [39]. Three sections/sample (triplicates) were evaluated. Briefly sections were outlined and scanned at low magnification (×12.5). The thickness of each section was measured at higher magnification (×600) in three separate areas, and the average thickness of each section was calculated. Cells were counted under higher magnification (×600) using an oil immersion objective. Cell counts were estimated within a dissector height of 7 μm, using an 800 × 800 μm^2 ^grid size and a 60 × 60 μm^2 ^counting frame size. The coefficient of error was calculated based on the Gundersen equation [40]. ALDH1 staining was quantified using average number of ALDH1 positive cells divided by the average number of Hematoxylin counterstained cells in each group and expressed as % mean ± standard deviation (SD).

### Western blot and densitometry analysis

Total protein was extracted from tissue and separated by one-dimensional Western blot using 10% gradient Tris-HCl gels (Bio-Rad, Hercules, CA; 10 μg total protein/lane) using standard procedures as described previously [1]. Proteins were transferred to a nitrocellulose membrane (0.45 μm; Bio-Rad). Recombinant ALDH1A1 (rALDH1; 1 μg/lane) produced in collaboration with Dr. Jim Dias (University of Albany, Albany, NY) was used as a positive control. Mouse anti-human ALDH1 (1:2000; clone 44, BD Transduction Lab San Jose, CA) and peroxidase-conjugated donkey anti-mouse IgG (1:5000; Jackson ImmunoResearch Laboratories, West Grove, PA) antibody was used to detect ALDH1. Human β-actin was used as a loading control and was detected with mouse anti-actin (1:2000; Sigma, St. Louis, MO). Antibodies were diluted in Blocker solution (Sigma) containing 0.05% Tween 20 (Bio-Rad). The membranes were washed after each step using Tris-buffered saline (10 mM Tris and 0.15 M NaCl, pH7.5) containing 0.05% Tween 20. The protein bands were detected using SuperSignal West Dura substrate (Thermo Scientific, Rockford, IL). MagicMark XP Western standards (Invitrogen, Carlsbad, CA) were used to estimate molecular weight. Digital images were obtained with a Chemidoc XRS Imaging System (BioRad) and analyzed by Quantity One software (Bio-Rad) according to manufacturer's recommendation. The relative density of each ALDH1 band was expressed as a ratio of the density of ALDH1 band and the corresponding β-actin band.

### Assessment of ALDH1 expression and enzyme activity by flow cytometry

The tissue was dissociated mechanically and enzymatically using a solid human tissue dissociation protocol (Stemcell Technologies, Vancouver, BC) with minor modifications. In brief, tissue was minced, washed in cold Dulbecco's Phosphate Buffered Saline (DPBS; Invitrogen, Carlsbad, CA) and suspended in Dulbecco's Modified Eagle Medium/Nutrient Mixture F-12 (DMEM/F12; Invitrogen) supplemented with 5% Fetal Bovine Serum (FBS; Invitrogen), collagenase type I (Worthington, Lakewood, NJ) and DNase 1 (Stemcell Technologies) followed by incubation with gentle agitation (2 hours; 37°C). The cell pellet and tissue fragments were separated by centrifugation (5 minutes; 100 × g) followed by a wash with DPBS. A single-cell suspension was obtained after filtering through 40 μm sterile nylon mesh (BD Falcon, San Jose, CA). The flow through was collected in a fresh tube, centrifuged (5 minutes; 100 × g), washed and suspended in DPBS. To remove and lyse red blood cells the cells were treated with ammonium chloride solution (BioLegend, San Diego, CA; 10 minutes; 4°C). Cells were then suspended in DPBS with 2% BSA and the cell count was determined using a Coulter Counter (Beckman, Brea, CA).

Aldehyde dehydrogenase enzyme activity in viable cells was determined using a fluorogenic dye based ALDEFLOUR assay (Stemcell Technologies) according to the manufacturer's instructions. In brief, cells were suspended (0.5 × 10^6 ^cells/mL) in ALDEFLUOR assay buffer containing ALDH substrate (Bodipy-Aminoacetaldehyde) and incubated (45 minutes; 37°C). As a reference control, the cells were suspended in buffer containing ALDEFLUOR substrate in the presence of diethylaminobenzaldehyde (DEAB), a specific ALDH1 enzyme inhibitor. Propidium iodide (2 μg/mL; Sigma, St. Louis, MO) was used to exclude dead cells. The cells were analyzed using a FACSCalibur flow cytometer (BD Biosciences, Rockville, MD) and the data was analyzed using FlowJo 7.6.1 software (Tree Star, Ashland, OR).

### Statistical Analysis

The outcome variables were expressed as mean ± SD. SPSS (Student version 7.5, SPSS Inc., Chicago, IL) was used for statistics. The independent samples t-test was used to test the statistical difference between groups. Correlation was analyzed by calculating a Pearson correlation coefficient (r). P values < 0.05 were considered statistically significant.

## Results

### ALDH1 mRNA expression

**Figure 1:**
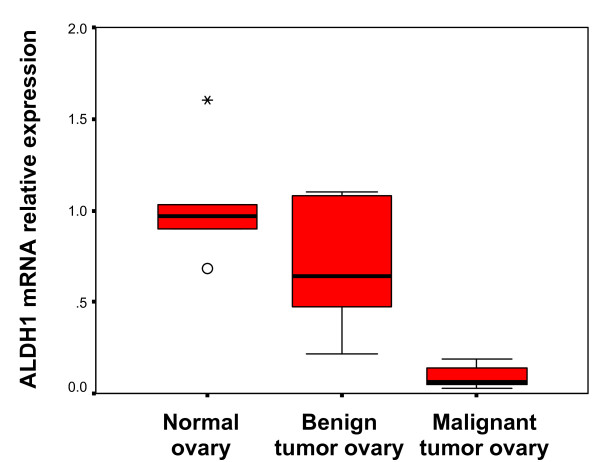
**ALDH1 mRNA expression differs among normal ovary, benign tumors and malignant tumors**. ALDH1 mRNA levels determined by qRT-PCR were significantly lower in malignant tumors than in normal ovary and benign tumors. ALDH1 mRNA did not significantly differ between benign tumors and normal ovary. Values for ALDH1 were normalized to actin as an internal control. The boxplots represent the median (dark horizontal line), range (whiskers), and 25th-75th percentile (box) for each group (n = 5/group).

ALDH1 mRNA expression was significantly lower in malignant ovarian tumors (n = 5) compared to normal ovary (p < 0.001; n = 5) and benign ovarian tumors (p = 0.008; n = 5) (Figure 1). There was no significant difference in ALDH1 mRNA expression between normal ovary and benign ovarian tumors (p = 0.18). The target amplified gene was confirmed as *ALDH1A1 *[GenBank: NM_000689.3] (data not shown).

### ALDH1 protein expression and localization

**Figure 2:**
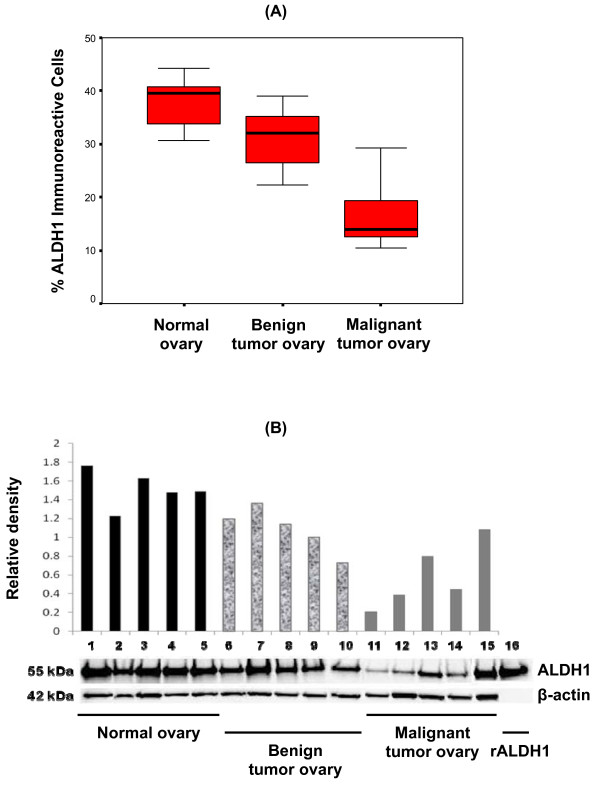
**ALDH1 protein expression differs among normal ovary, benign tumors and malignant tumors**. [A] The number of ALDH expressing cells was significantly lower in malignant tumors than in normal ovary and benign tumors. The boxplots represent the median (dark horizontal line), range (whiskers), and 25th-75th percentile (box) for each group (n = 5/group). Quantification of ALDH1+ cells was performed using StereoInvestigator software. [B] Protein was detected in tissue homogenates by Western blot (10 μg protein/lane). A single immunoreactive band reacted with mouse anti-ALDH1 (upper panel). Recombinant ALDH1 (1 μg; lane 16) was used as a positive control. Human actin was used as a loading control (lower panel). Densitometry analysis confirmed differential ALDH1 protein expression in ovarian tissues. Each sample was plotted on Y-axis as ratio of the relative density of ALDH1 normalized to actin.

The proportion of ALDH1 immunostained cells was significantly lower in malignant ovarian tumors (17.1 ± 7.6%; n = 5) compared to normal ovaries (37.4 ± 5.4%; p = 0.001; n = 5) and benign ovarian tumors (31.0 ± 6.7%; p = 0.015; n = 5) (Figure 2A). There was no significant difference between normal ovary and benign ovarian tumors (p = 0.11), thus confirming the mRNA data. The ALDH1 mRNA expression levels and the proportion of immunostained cells was positively correlated (r = 0.7; p < 0.01).

ALDH1 protein was detected as a single band at 55 kDa in all of the ovarian tissues tested by Western blot (Figure 2B). Densitometry analysis of the blots showed lower levels of ALDH1 in malignant tumors compared to normal ovary and benign tumors. Furthermore, a higher ALDH1 band intensity was detected in a well differentiated malignant tumor (lane 15; Figure 2B) compared to poorly differentiated tumors (lane 11 -14; Figure 2B). A strong positive correlation was observed between the levels of ALDH1 protein expression in Western blot and proportion of ALDH1 immunostained cells (r = 0.8; p < 0.01) among the tested samples.

**Figure 3:**
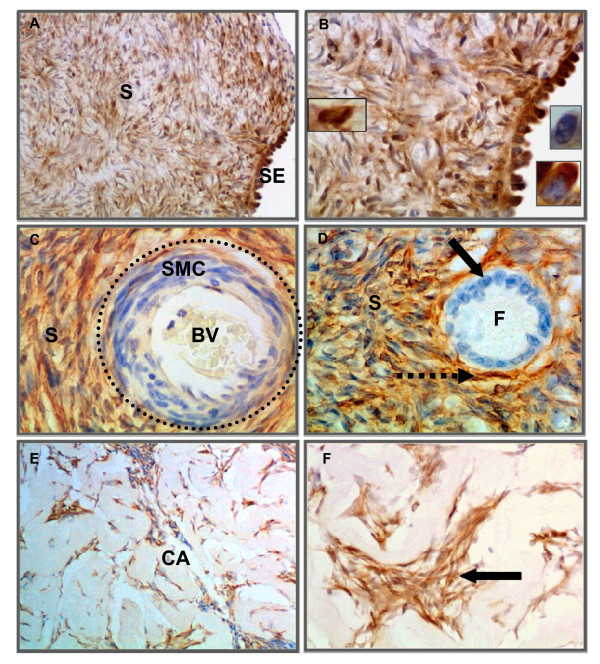
**Immunohistochemical localization of ALDH1 in normal ovaries**. [A-B] Intense staining of numerous cells of stroma (S) and surface epithelial cells (SE) was observed in normal ovary. Insets showing examples of ALDH1 stained stromal and epithelial cells and an example of an occasional unstained epithelial cell at high magnification (×1000). [C] ALDH1 staining was absent in smooth muscle cells (SMC; dotted outline) surrounding blood vessel (BV) and [D] in the granulosa cell layer (black arrow) lining follicles (F). However, the theca layer (dotted arrow) and neighboring stromal cells (S) expressed ALDH1. [E-F] Representative images of ALDH1 stained cells (arrow; fibroblast like cells) within the corpus albicans (CA). Sections were counterstained with hematoxylin. (Original magnifications: ×200, ×400, ×400, ×400, ×100 and ×400 respectively).

ALDH1 immunostaining was observed in various cell types in normal ovary and serous ovarian tumors. In normal ovary, a diffuse ALDH1 staining pattern was observed in the stroma in fibroblasts-like cells and fibrous tissue. In addition, the surface epithelial cells stained intensely although there were occasional cells without stain (Figure 3A and 3B). The smooth muscle cells surrounding the blood vessels and the granulosa cell layer surrounding developing follicles did not stain for ALDH1; however, the stromal cells in the perivascular regions and in the developing theca layer of follicles showed ALDH1 staining (Figure 3C and 3D). The fibrous tissue between cords of luteal cells in the regressing corpus luteum (corpus albicans) also stained for ALDH1 but not the cells of corpus luteum (Figure 3E and 3F).

**Figure 4:**
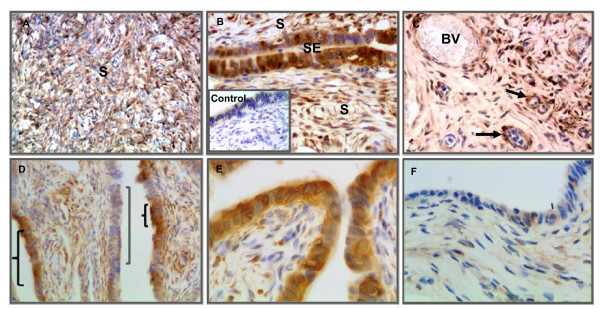
**Immunohistochemical localization of ALDH1 in benign serous ovarian tumors**. [A] Uninvolved regions adjacent to benign tumors have a similar staining pattern as normal ovary. [B] Intense staining was observed in stromal (S) and surface epithelial (SE) cells of benign tumors. The inset shows a primary antibody control (anti-ALDH1 omitted). [C] The ALDH1 staining pattern surrounding neo-angiogenic blood vessels (arrow) in a benign tumor ovary differs from normal ovary. [D] Discontinuous pattern of ALDH1 expression was observed along the surface epithelium of benign tumor projections. [E] ALDH1 staining was predominantly expressed in surface epithelial cells of serous papillary projections with less apparent staining of stroma compared to uninvolved areas adjacent to benign tumors. [F] A representative example from the same tissue sample as in E with little or no ALDH1 expression. Sections were counterstained with hematoxylin. (Original magnifications: ×200, ×400, ×400, ×200, ×400 and ×400 respectively).

The staining pattern of ALDH1 in uninvolved areas adjacent to benign serous ovarian tumors was similar to that of normal ovary (Figure 4A). In contrast to normal ovary, strong ALDH1 expression was observed near some neo-angiogenic blood vessels in benign ovarian tumors (Figure 4C). In addition, staining of the surface epithelium was patchy compared to normal ovary (Figure 3A) and contained areas of intense staining adjacent to areas of no staining (Figure 4D - 4F).

**Figure 5:**
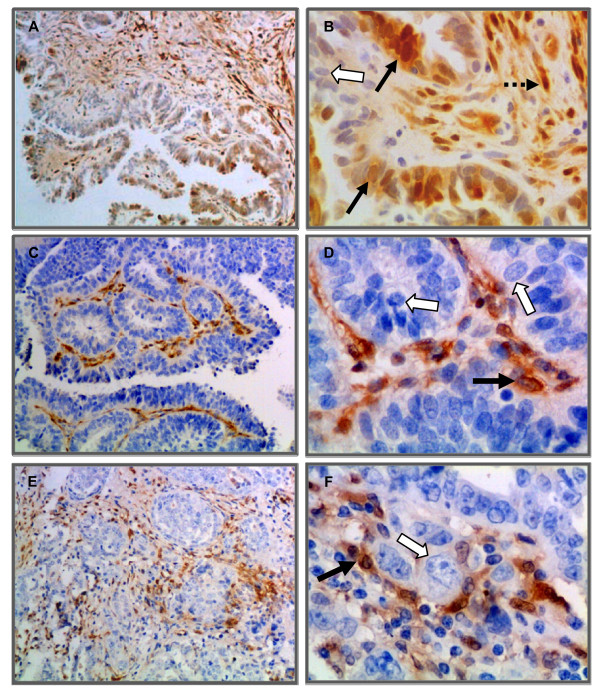
**Immunohistochemical localization of ALDH1 in malignant serous ovarian tumors**. ALDH1 expression was heterogeneous in malignant tumors. [A-B] A well differentiated tumor showing ALDH1 expression in numerous cells of epithelium and stroma with varying staining intensities (black arrows) or no staining (white arrow). Note: the nuclei are small, regular and lack prominent nucleoli, which is characteristic of a low grade tumor. [C-D] A poorly differentiated tumor showing absence of ALDH1 expression in tumor epithelial cells (white arrows). A few adjacent tumor stromal cells (black arrow) expressed ALDH1. [E-F] Representative images of ALDH1 staining in a poorly differentiated tumor. Characteristic tumor cells with activated nucleus (white arrow) show no ALDH1 expression, while adjacent stromal tissue contained few ALDH1+ cells (black arrow). Sections were counterstained with hematoxylin. (Original magnifications: ×100, ×400, ×100, ×400, ×100 and ×400 respectively).

**Figure 6:**
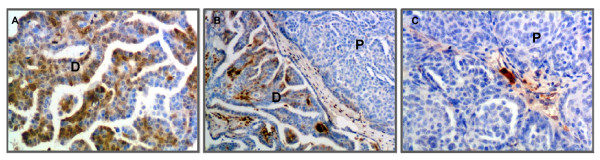
**Expression of ALDH1 was absent in regions with poorly differentiated tumor cell morphology while adjacent differentiated regions were highly stained**. [A] Numerous cells expressed ALDH1 in differentiated tumor regions (D) with micro-papillary architecture. [B] Representative section with adjacent areas showing strikingly different ALDH1 expression in differentiated and poorly differentiated regions. [C] Reduced or absent ALDH1 expressing cells in poorly differentiated regions (P) with solid tumor cell nests. Sections were counterstained with hematoxylin. (Original magnifications: ×200, ×100 and ×200 respectively).

In malignant serous ovarian tumors ALDH1 staining varied (strong to weak or no staining; Figure 5) and was seen primarily in the fibroblast like cells in the stroma and a few well differentiated tumor epithelial cells (Figure 5B). Well differentiated malignant tumor cells (Figure 5A - 5B) showed higher ALDH1 expression compared to poorly differentiated tumor cells (Figure 5C - 5F). Interestingly, ALDH1 staining differed in the same malignant tumor tissue based on cellular differentiation (Figure 6). Poorly differentiated regions of solid tumor cell nests (Figure 6B and 6C) had little or no ALDH1 expression compared to the adjacent, highly stained differentiated regions with micro-papillary tumor architecture (Figure 6A and 6B).

**Figure 7:**
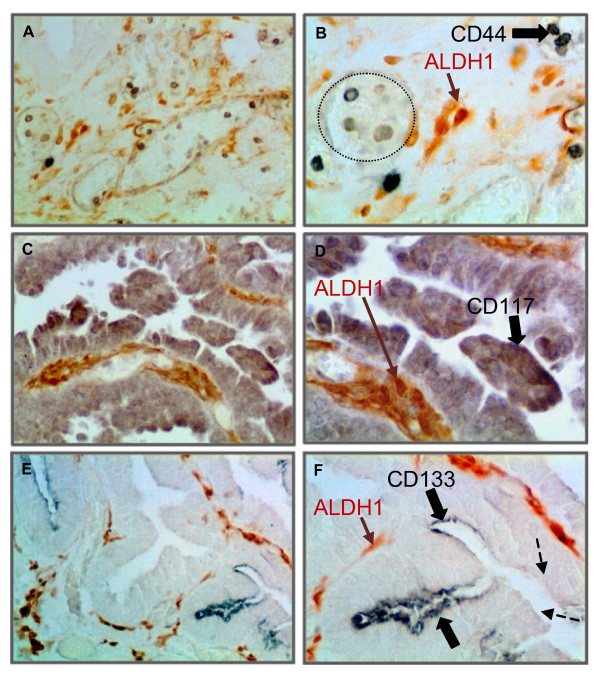
**ALDH1 and cancer stem cell (CSC) markers are expressed in different cell populations in malignant ovarian tumors**. [A-B] shows ALDH1 (brown) and CD44 (black) immunostaining in different cells in the tumor stroma. Representative image showing CD44+ cells (presumptively blood cells) primarily localized in or near blood vessels (dotted line). [C-D] shows localization of ALDH1 (brown) and CD117 (black) immunostaining in different cells. CD117+ cells were exclusively localized in the tumor epithelium. [E-F] shows ALDH1 (brown) and CD133 (black) immunostaining in different cells. CD133+ cells were localized to the tumor apical surface of epithelial cells in discontinuous patches of stained (solid arrows) and adjacent unstained cells (dotted arrows). Sections were not counterstained. (Original magnifications: ×200, ×400, ×200, ×400, ×200 and ×400 respectively).

To further investigate ALDH1 expression in high grade malignant serous ovarian tumors, sections were co-stained with CD44, CD117 and CD133 to determine if there was an association with CSC markers (Figure 7). ALDH1 and CSC markers were expressed in different cell populations. CD44 was expressed in lymphocytes in and near blood vessels as expected. CD117 (Figure 7C &7D) and CD133 (Figure 7E &7F) expression was localized in tumor epithelial cells while ALDH1 immunostaining occurred in the tumor stroma. We evaluated sections from 3 poorly differentiated malignant ovarian tumors and found occasional cells (< 1%) that were co-stained with ALDH1 and CD44. However, it is not clear whether they were tumor cells or infiltrating lymphocytes. We did not find any visible co-staining with ALDH1 and CD117 or CD133 markers.

### ALDH1 enzyme activity in ovarian cells

**Figure 8:**
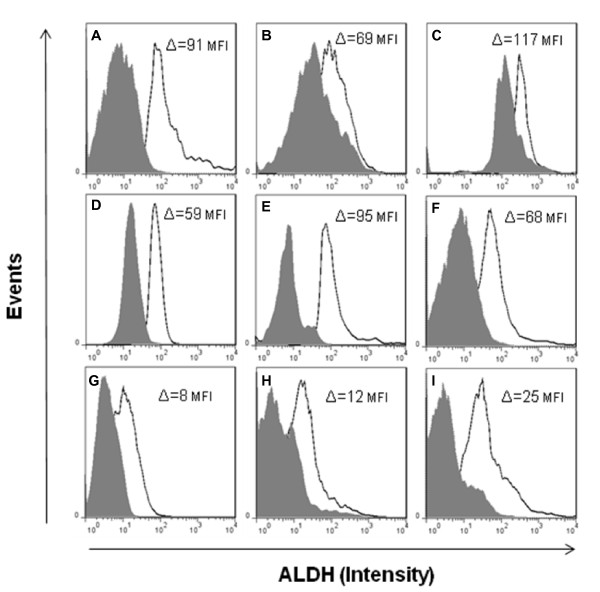
**Flow cytometry analysis of normal ovarian cells, benign and malignant tumor cells with ALDEFLUOR**. The ALDH1 enzyme activity was calculated as a difference (Δ) in mean fluorescence intensity (MFI). The background in the presence of the ALDH1 inhibitor DEAB (shaded gray) was subtracted from the mean fluorescence intensity of cells incubated with ALDEFLUOR alone (black line) for each cell preparation. Normal ovarian cells (A-C; Δ = 92.3 ± 24 MFI) and benign tumor cells (D-F; Δ = 74 ± 18.7 MFI) show higher ALDH1 enzyme activity than malignant tumor cells (G-I; Δ = 15 ± 8.8 MFI) as depicted in the overlay histogram plots.

The mean fluorescence intensity (MFI) was significantly decreased in malignant ovarian tumors (15 ± 8.8 MFI; n = 3) compared to normal ovary (92.3 ± 24 MFI; p = 0.02; n = 3) and benign ovarian tumors (74 ± 18.7 MFI; p = 0.018; n = 3) (Figure 8). While no significant difference in MFI was observed between normal ovary and benign tumors (p = 0.3). In addition, the proportion of ALDH^Bright ^cells was lower in malignant ovarian tumors (6.4 ± 2.9%) compared to normal ovary (22.8 ± 6.4%) and benign tumors (16.3 ± 5.6%). The ALDEFLUOR assay was positively correlated with the proportion of cells expressing ALDH1 by semi-quantitative immunohistochemistry (r = 0.77; p < 0.01). Overall, the estimation of enzyme activity in ovarian cells was consistent with ALDH1 mRNA and protein expression levels.

## Discussion

In summary, the ALDH1 expression and enzyme activity was lower in malignant ovarian tumors compared to normal ovary, while benign ovarian tumors exhibited expression levels slightly lower but similar to normal ovaries. This is strikingly different than in breast, lung or colon cancers in which ALDH1 expression is limited in the normal tissue but is significantly increased in malignant tissue [8,10,36].

Our results are consistent with studies using gene expression microarrays which showed that the *ALDH1A1 *gene was down-regulated in malignant ovarian tumors compared to benign ovarian tumors [41,42] or to normal ovary [43,44]. This is the first report which compares ALDH1 expression and enzyme activity in normal ovary and serous ovarian tumors in one study. ALDH1 was localized in surface epithelial cells and stroma in the cortical and medullary regions of normal ovary and was not evident within follicles or blood vessel endothelial cells. The widespread and high expression in normal ovary is consistent with studies which suggest that ALDH1 has an obligatory functional role in normal ovarian physiology. [27,28]

The ALDH1 protein expression and enzyme activity were correlated. However, the proportion of ALDEFLUOR positive cells (ALDH^Bright^) was smaller than the proportion of ALDH1 immunostained cells suggesting that not all ALDH1 may be active. This was also observed by Deng et al. [7].

Our study also shows for the first time that ALDH1 expression in malignant serous ovarian tumors is heterogeneous and the localization appears to be based on the level of cellular differentiation. It is known that patients with well differentiated (low-grade) malignant ovarian tumors have a higher survival rate than patients with less differentiated (high-grade) tumors [45]. ALDH1 staining was substantially lower in less differentiated tumor cells compared to differentiated tumor cells. Since the degree of morphological differentiation is associated with malignant potential, this suggests a potential relationship to clinical outcomes. The higher expression of ALDH1 in benign tumors without malignant potential is congruent with this observation. It is also interesting to note that low-grade tumors show poor responses to chemotherapy compared to high-grade tumors [46]. This is thought to be due to more rapid metabolism of chemotherapeutics which could be correlated with our observation of higher ALDH1 expression in low-grade tumors. Thus, further studies are warranted to assess the possibility that ALDH1 expression could be used in pathology evaluation of tissue histology to predict disease prognosis and response to chemotherapy in ovarian cancer.

Previous studies showed that higher ALDH1 expression in tumor cells is associated with poor clinical outcomes in breast, [36,47] lung, [10,48] colon [8] cancer patients. However, Chang et al. reported that higher ALDH1 expression in tumor cells was correlated with a favorable patient prognosis in ovarian cancer [24]. They examined the relationship of ALDH1 levels to survival in ovarian cancer patients and did not analyze the histological subtypes of ovarian tumors separately. In contrast, Deng et al. observed that a relatively high number of ALDH1 expressing tumor cells in malignant serous ovarian tumors was correlated with poor survival [7]. These contrasting clinical outcome observations in ovarian cancer could be due to a number of factors including differences in cell counting methodology and differences in the tumor types in the study groups. Although we did not examine the relationship of ALDH1 to survival (the data was not available), the association of very low or no ALDH1 expression with poorly differentiated tumors is consistent with the concept that loss of ALDH1 is associated with an aggressive tumor type. This is also consistent with our finding that ALDH1 expression in benign serous tumors (without malignant potential) is similar to normal ovary. Thus, our conclusion is similar to Chang et al., [24] even though our study was restricted to serous ovarian tumors similar to Deng et al. [7]. Another design difference among the three studies was that in our study we tabulated total ALDH1 immunostained cells, whereas stromal immunostaining of ALDH1 was excluded in the other studies. A strength of our study is that ALDH1 expression was evaluated using three analytical methods and used immunohistochemistry to demonstrate differences in ALDH1 distribution.

Recent observations suggest ALDH1 is a marker for CSCs in various malignancies and that ALDH1 in CSCs is associated with chemoresistance and increased malignant potential [7-9,36,37,47,48]. Chute et al. demonstrated that ALDH1 enzyme activity is necessary for human hematopoietic stem cell (HSC) differentiation, and inhibition of this enzyme results in expansion of HSCs [49]. In solid tumors the identity of CSCs [50] and the role of ALDH1 is less clear [51]. Stem cells in the human ovary are involved in ovarian development, normal function and it has been suggested they have a role in pathological conditions such as infertility and ovarian cancer [52,53]. Putative CSCs isolated from ovarian cancer cell lines [54], ascites [54,55] and primary ovarian tumor tissues [56-58] displayed CSC growth characteristics. Emerging evidence in ovarian cancer suggests that cells expressing CD44, [56,57] CD117 [57] or CD133 [58] cell-surface markers have CSC properties. However, the identification of CSC and their molecular characteristics, as well as the clinical significance of an ovarian CSC phenotype is not yet clear. We found that ALDH1 appears to be expressed in different cell populations than CD44, CD117 and CD133. However, a caveat is that we did not examine the entire tumor since parts of the tumor are retained by the pathologist for diagnostic evaluation. Thus it is possible that the CSC might be in another area of the tumor that was not sampled. In preliminary studies we examined co-expression of ALDH1 and the CSC markers by flow cytometry and did not find a consistent pattern of association. Although we cannot conclude that ovarian CSCs do not contain ALDH1, this initial examination suggests differences from other solid tumors [8,36,37]. Thus, our findings and previous studies suggest that ALDH1 may not be an ideal marker for isolating CSCs in ovarian cancer. However, these findings remain to be confirmed.

## Conclusions

We found that the total ALDH1 expression is significantly reduced in malignant serous ovarian tumors compared to normal ovaries and that expression in benign serous ovarian tumors is similar to normal ovary. ALDH1 was expressed in malignant tumor cells but at a low level and was absent in the more aggressive poorly differentiated malignant tumor cells. The heterogeneity of ALDH1 expression pattern suggests ALDH1 could be used as a novel indicator of prognosis and possibly as an indicator of responses to chemotherapy. Further investigation could facilitate understanding the role of ALDH1 in the ovary and ovarian tumors.

## Competing interests

The authors declare that they have no competing interests.

## Authors' contributions

KP, JL and SE worked to develop the experimental design. KP performed the experiments, statistical calculations and wrote the manuscript. SE facilitated the PCR and gene sequencing, and assisted KP in data analysis. AB developed the immunohistochemistry (IHC) protocols, facilitated tissue collection and assisted in writing the manuscript. MB assisted with tissue collection and IHC tissue processing. JL conceived the study, mentored KP in scientific methods and data analysis and assisted in drafting and finalizing the manuscript. All authors approved the final version of the manuscript.
